# A nomogram for predicting echocardiogram prescription in outpatients: an analysis of the NAMCS database

**DOI:** 10.3389/fcvm.2023.1183504

**Published:** 2023-10-16

**Authors:** Yujian Liu, Yanhan Deng, Hongjie Wang, Wanjun Liu, Xingwei He, Hesong Zeng

**Affiliations:** ^1^ Division of Cardiology, Department of Internal Medicine, Tongji Hospital, Tongji Medical College, Huazhong University of Science and Technology, Wuhan, China; ^2^ Hubei Provincial Engineering Research Center of Vascular Interventional Therapy, Wuhan, China; ^3^ Department of Rheumatology and Immunology, Tongji Hospital, Tongji Medical College, Huazhong University of Science and Technology, Wuhan, China

**Keywords:** echocardiogram, outpatient, nomogram, predictor, propensity score matching

## Abstract

**Background and objective:**

Cardiovascular disease is the leading cause of morbidity and mortality globally. Echocardiography is a commonly used method for assessing the condition of patients with cardiovascular disease. However, little is known about the population characteristics of patients who are recommended for echocardiographic examinations.

**Methods:**

The National Ambulatory Medical Care Survey was a cross-sectional survey previously undertaken in the USA. In this study, publicly accessible data from the National Ambulatory Medical Care Survey database (for 2007–2016 and 2018–2019; data for 2017 was not published) were utilized to create a nomogram based on significant risk predictors. The study was performed in accordance with the relevant guidelines and regulations stipulated in the National Ambulatory Medical Care Survey database. Patients were randomly assigned to one of two groups: training cohort or validation cohort. The latter was used to assess the reliability of the prediction nomogram. Decision curve analysis was performed to evaluate the net benefit. Propensity score matching analysis was used to evaluate the relevance of echocardiography to clinical decision-making.

**Results:**

A total of 217,178 outpatients were enrolled. Multivariable logistic regression analysis demonstrated that hypertension, hyperlipidemia, coronary artery disease/ischemic heart disease/history of myocardial infarction, congestive heart failure, major reason for visit, metropolitan statistical area, cerebrovascular disease/history of stroke or transient ischemic attack, previously assessed, insurance, referred, diagnosis, and reason for visit were all predictors of echocardiogram prescription in outpatients. The reliability of the predictive nomogram was confirmed in the validation cohort. After propensity score matching, there was a significant difference in new cardiovascular agent prescriptions between the echocardiogram and no echocardiogram groups (*P* < 0.01).

**Conclusion:**

In this cohort study, a nomogram based on the characteristics of outpatients was developed to predict the possibility of prescribing echocardiography. The echocardiogram group was more likely to be prescribed new cardiovascular agents. These findings may contribute to providing information about the gap between actual utilizations and guidelines and the actual outpatient practice, as well as meeting the needs of outpatients.

## 1. Introduction

Cardiovascular disease (CVD) is the leading cause of morbidity and mortality globally (1). The growing global burden of CVD jeopardizes the completion of the Global Action Plan for the Prevention and Control of Non-communicable Diseases (NCDs), which aims to reduce premature NCD-related mortality by 25% by 2025 (2). Reducing the burden of CVD and delivering these high-priority goals in diverse social and economic settings among heterogeneous populations pose enormous challenges for healthcare systems (3).

Echocardiography is the primary imaging modality to diagnose cardiac conditions (4). Imaging techniques such as Doppler tissue imaging, speckle tracking, and real-time 3-dimensional echocardiography are projected to enable a comprehensive assessment of the heart’s structure and function in the not-too-distant future (5, 6). Exercise, dobutamine, adenosine, or dipyridamole stress echocardiography can be used to assess coronary artery disease (5, 6). Additionally, physicians will be able to better grasp the status of patients with CVD and formulate more appropriate treatment strategies, resulting in fewer unnecessary admissions (7–9).

However, little is known about the demographics of patients who are recommended for echocardiographic examinations. In this study, using publicly available data from the National Ambulatory Medical Care Survey (NAMCS), we sought to describe the characteristics of this population, establish a nomogram for predicting echocardiogram prescription, and describe the relationship between echocardiogram prescription and the use of new cardiovascular agents.

## 2. Methods

### 2.1. Study design and population

The NAMCS was a cross-sectional, two-stage sample survey conducted in the USA by the National Center for Health Statistics, a Centers for Disease Control and Prevention unit. The NAMCS database comprises responses of non-federally employed ambulatory physicians to a standardized survey instrument. The survey protocol was approved by the Research Ethics Review Board of the National Center for Health Statistics and written informed consent was obtained from all the participants. The form includes fields for demographic data, diagnoses, and medications. The keying and coding of survey data are performed by trained hospital staff, are subject to internal quality control procedures, and have an error rate of 0%–1% (National Ambulatory Medical Care Survey: Centers for Disease Control and Prevention, National Center for Health Statistics: Ambulatory health care data. Available at: https://www.cdc.gov/nchs/ahcd/index.htm. Accessed November 29, 2022).

**Figure 1:**
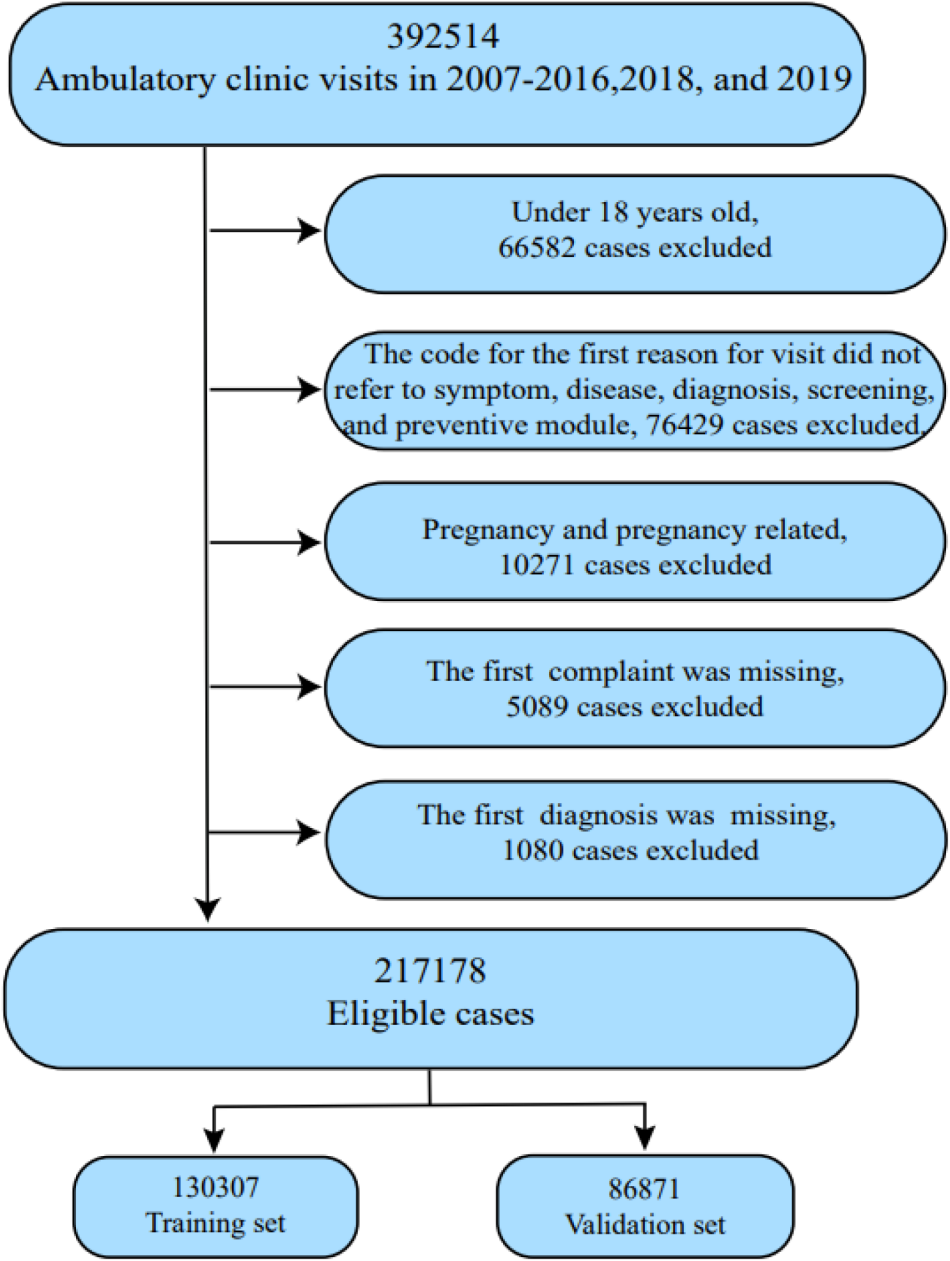
Flow chart for patient selection.

Publicly available non-personally identifiable data for outpatients were used in this study. The study was performed in accordance with the relevant guidelines and regulations stipulated in the NAMCS database and included data from 2007 to 2016 and 2018 to 2019 (*n *= 332,814) to generate reliable national estimates of patients prescribed an echocardiogram. The exclusion criteria were as follows: patients under the age of 18 years (*n *= 66,582); patients whose codes for reason for a visit were treatment module, injury, adverse effect module, test results module, administrative module, entries that were not codable, and blank entries (*n *= 76,429); patients involved in pregnancy and pregnancy-related events (*n *= 10,271); and patients whose records were incomplete (6,169). Eventually, a total of 217,178 patients were enrolled, and were randomly assigned to a training cohort (60%, *n *= 130,307) and a validation cohort (40%, *n *= 86,871) (Figure 1). As pregnancy and pregnancy-related patients are highly diverse from the general population, we analyzed data separately (Supplementary Table S1). Given the limited number of pregnant patients prescribed echocardiograms in this database, more studies are needed to interpret the findings.

### 2.2. Clinical variables and definition

The variables included age, sex, race, insurance means/expected sources of payment for the visit (self-pay was designated as “no insurance” and the other sources were designated as “yes”), tobacco use, reasons for visit (if the first three reasons were related to heart disease, the reason for visit was designated as “heart disease related”, otherwise it was designated as “other”), major reason for visit (new problem was designated as “new problem” and chronic problem/routine, chronic problem/flare-up, pre-/post-surgery, and preventive care were designated as “other”), whetherpatient was referred for this visit (designated as “referred”), whether the patient attended the practice before (designated as “previously assessed”), diagnosis (whether the first three diagnoses were cardiac disease), cerebrovascular disease/history of stroke or transient ischemic attack (designated as CEBVD), congestive heart failure (CHF), coronary artery disease/ischemic heart disease/history of myocardial infarction (designated as “CAD”), diabetes, hyperlipidemia, hypertension, obesity, chronic obstructive pulmonary disease (COPD), chronic kidney disease (CKD), end-stage renal disease (ESRD), metropolitan statistical area (MSA), and new cardiovascular agents prescribed (designated as “NCAP”) (from RX1 to RX8). The cardiovascular medication codes were 040–056, 081–083, 154–158, 173, 174, 211, 212, 241, 252, 261, 262, 283, 285, 316, 317, 274, 275, 303, 319, 325, 340, 342, 396, 430, and 433 (Supplementary Table S2). The cardiac disease-related codes for the reason for the visit were 10351, 10500, 10502, 10503, 12600–12700, 14150, 14200, 25050–25200, 25500, 25300, 29500, 31000, 32350, and 33700. We used the Clinical Classifications Software (CCS) map to International Classification of Diseases-9-CM (ICD-9-CM) codes and ICD-10-CM, followed by the extraction of patients diagnosed with cardiac disease (10, 11). CKD and ESRD were not included in the analysis, except for baseline data, because only 2014–2019 data was available in the NAMCS (Supplementary Tables S3–S5).

### 2.3. Statistical analysis

Pearson’s chi-squared test or the *t*-test was used to compare differences between two groups (echocardiogram group vs. no echocardiogram group, training cohort *vs*. validation cohort). For variables with <30% missing values (missing ratios: insurance, 5.3%; referred, 12.6%; major reason for visit, 2.6%; diagnosis, 0.2%; tobacco use, 27.1%), missing data were filled in using multiple imputation. Patient characteristics were presented as mean ± SD or frequency and percentage, as appropriate. Potential candidate variables were screened using least absolute shrinkage and selection operator (LASSO) regression with 10-fold cross-validation followed by a multivariable logistic regression analysis of the variables derived from LASSO regression. Variables with a *P*-value < 0.05 were included in the nomogram model. A nomogram for predicting the probability of prescribing an echocardiogram was subsequently constructed based on the results from the final regression analysis. A visual calibration curve comparing the apparent probability, bias-corrected probability, and ideal probability curves was generated to show the calibration of the prediction model. Decision curve analysis (DCA) was undertaken to assess the clinical usefulness of the prediction model. Propensity score matching (PSM) was used to limit possible selection deviation between the two groups and was carried out as a 1:3 ratio nearest neighbor matching. The following variables were included: age, sex, race, insurance, reason for visit, major reason for visit, referred, tobacco use, previously assessed, diagnosis, CEBVD, CHF, CAD, diabetes, hyperlipidemia, hypertension, obesity, COPD, and MSA. *P*-values < 0.05 were considered significant. All statistical analyses were performed using R software (version 4.1.2) with the dplyr, pROC, glmnet, mice, caret, rms, rmda, ResourceSelection, ggplot2, forestplot, and MatchIt packages.

## 3. Results

### 3.1. General characteristics and outcomes

A total of 217,178 patients for whom relevant data were available from 2007 to 2016 and 2018 to 2019 (data for 2017 were not available) were included. Patients were divided into an echocardiogram group (*n *= 2,973) and a no-echocardiogram group (*n *= 214,205). Significant differences in age, sex, race, tobacco use, insurance, reason for visit, referred, previously assessed, diagnosis, CEBVD, CHF, CAD, diabetes, hyperlipidemia, hypertension, obesity, and MSA were detected between the two groups (Table 1). However, no differences in patient characteristics were observed between the training and validation cohorts, except for tobacco use (Supplementary Table S6).

**Table 1:** Characteristics at baseline between the echocardiogram and the no echocardiogram groups.

Variable	Total (*n *= 217,178)	No echocardiogram (*n *= 214,205)	Echocardiogram (*n *= 2,973)	*P*-value
Age mean ± SD	55.86 ± 18.09	55.77 ± 18.09	62.69 ± 16.19	<0.01
Sex *n* (%)				<0.01
Male	127168 (58.55)	125561 (58.62)	1607 (54.05)	
Female	90010 (41.45)	88644 (41.38)	1366 (45.95)	
Race *n* (%)				<0.01
White	186282 (85.77)	183811 (85.81)	2471 (83.11)	
Black	20526 (9.45)	20175 (9.42)	351 (11.81)	
Other	10370 (4.77)	10219 (4.77)	151 (5.08)	
Tobacco use *n* (%)				<0.01
No	182556 (84.06)	179985 (84.02)	2571 (86.48)	
Yes	34622 (15.94)	34220 (15.98)	402 (13.52)	
Insurance *n* (%)				<0.01
No	18305 (8.43)	18215 (8.50)	90 (3.03)	
Yes	198873 (91.57)	195990 (91.50)	2883 (96.97)	
Reason for visit *n* (%)				<0.01
Other	178136 (82.02)	177420 (82.83)	716 (24.08)	
Heart disease-related	39042 (17.98)	36785 (17.17)	2257 (75.92)	
Referred *n* (%)				<0.01
No	157425 (72.49)	155746 (72.71)	1679 (56.47)	
Yes	59753 (27.51)	58459 (27.29)	1294 (43.53)	
Previously assessed *n* (%)				<0.01
No	43759 (20.15)	42891 (20.02)	868 (29.20)	
Yes	173419 (79.85)	171314 (79.98)	2105 (70.80)	
Major reason for visit *n* (%)				0.56
Other	142923 (65.81)	140982 (65.82)	1941 (65.29)	
New problem	74255 (34.19)	73223 (34.18)	1032 (34.71)	
Diagnosis of heart disease *n* (%)				<0.01
No	185562 (85.44)	184810 (86.28)	752 (25.29)	
Yes	31616 (14.56)	29395 (13.72)	2221 (74.71)	
CEBVD *n* (%)				<0.01
No	212131 (97.68)	209360 (97.74)	2771 (93.21)	
Yes	5047 (2.32)	4845 (2.26)	202 (6.79)	
CHF *n* (%)				<0.01
No	12864 (98.01)	10173 (98.12)	2691 (90.51)	
Yes	4314 (1.99)	4032 (1.88)	282 (9.49)	
CAD *n* (%)				<0.01
No	05643 (94.69)	03484 (94.99)	2159 (72.62)	
Yes	11535 (5.31)	10721 (5.01)	814 (27.38)	
Diabetes *n* (%)				<0.01
No	187841 (86.49)	185469 (86.58)	2372 (79.78)	
Yes	29337 (13.51)	28736 (13.42)	601 (20.22)	
Hyperlipidemia *n* (%)				<0.01
No	177720 (81.83)	176018 (82.17)	1702 (57.25)	
Yes	39458 (18.17)	38187 (17.83)	1271 (42.75)	
Hypertension *n* (%)				<0.01
No	148339 (68.30)	147168 (68.70)	1171 (39.39)	
Yes	68839 (31.70)	67037 (31.30)	1802 (60.61)	
Obesity *n* (%)				<0.01
No	200580 (92.36)	198040 (92.45)	2540 (85.44)	
Yes	16598 (7.64)	16165 (7.55)	433 (14.56)	
COPD				<0.01
No	207919 (95.74)	205176 (95.78)	2743 (92.26)	
Yes	11535 (5.31)	10721 (5.01)	814 (27.38)	
CKD^a^				<0.01
No	52583 (97.28)	52038 (97.31)	545 (94.45)	
Yes	1469 (2.72)	1437 (2.69)	32 (5.55)	
ESRD^a^				0.99
No	53922 (99.76)	53346 (99.76)	576 (99.83)	
Yes	130 (0.24)	129 (0.24)	1 (0.17)	
MSA *n* (%)				<0.01
No	24159 (11.12)	23947 (11.18)	212 (7.13)	
Yes	193019 (88.88)	190258 (88.82)	2761 (92.87)	

CEBVD, cerebrovascular disease/history of stroke or transient ischemic attack; CHF, congestive heart failure; CAD, coronary artery disease/ischemic heart disease/history of myocardial infarction; MSA, metropolitan statistical area; COPD, chronic obstructive pulmonary disease; CKD, chronic kidney disease; ESRD, end-stage renal disease.

^a^ Only 2014–2019 data were analyzed.

### 3.2. Screening for predictive factors and nomogram development

**Figure 2:**
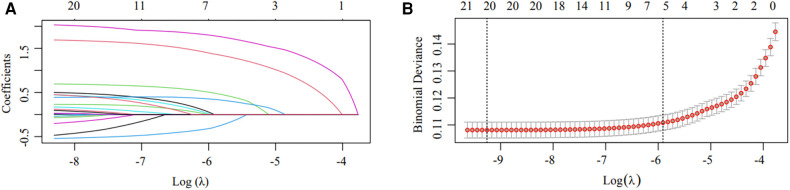
Candidate selection using the LASSO logistic regression model. (**A)** Tuning parameter (λ) selection using LASSO penalized logistic regression with 10-fold cross-validation. (**B)** LASSO coefficient profiles of the radiomic features. A coefficient profile plot was plotted versus log(λ). Each colored line represents the coefficient of one candidate.

**Figure 3:**
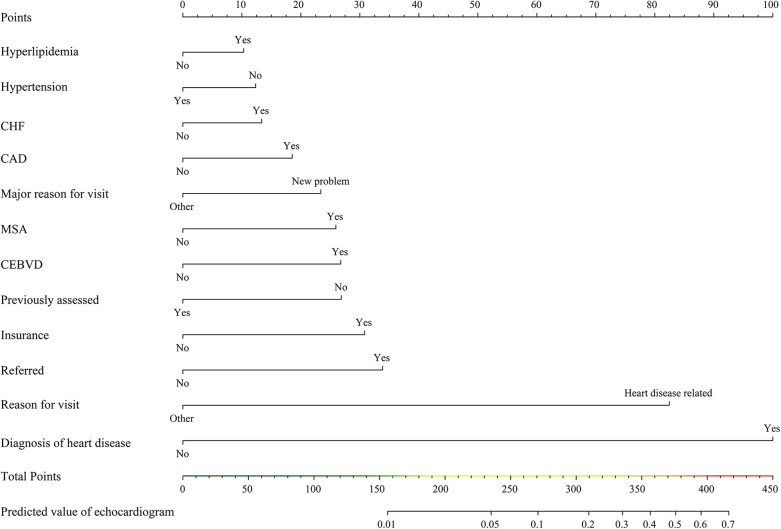
Nomogram for predicting echocardiogram prescription. CEBVD, cerebrovascular disease/history of stroke or transient ischemic attack; CHF, congestive heart failure; CAD, coronary artery disease/ischemic heart disease/history of myocardial infarction; MSA, metropolitan statistical area.

In the training cohort, LASSO regression was conducted for each candidate. The results of the univariate logistic regression analysis for all the candidates are shown in Supplementary Table S7. Subsequently, multivariable logistic regression analysis was conducted on the selected variables (Figure 2), with 12 predictors being included in the final model (Table 2). These 12 predictors were integrated into the nomogram (*R*^2 ^= 0.286, C-index = 0.892) (Figure 3). The Hosmer-Lemeshow test demonstrated that the model was a good fit (*P *= 0.228). For each patient, higher total points indicated a higher probability of being prescribed an echocardiogram.

**Table 2:** Echocardiogram multivariable logistic regression model in the training cohort.

Variable	Multivariable analysis based on the results of the LASSO regression analysis			Multivariable logistic regression model		
	OR	Lower CI	Upper CI	OR	Lower CI	Upper CI
Hypertension	0.74	0.66	0.84	0.77	0.69	0.87
Hyperlipidemia	1.24	1.11	1.38	1.24	1.11	1.39
CAD	1.47	1.29	1.67	1.47	1.30	1.67
CEBVD	1.71	1.40	2.09	1.75	1.43	2.13
CHF	1.26	1.05	1.51	1.32	1.10	1.58
Major reason for visit	1.64	1.46	1.83	1.63	1.45	1.83
MSA	1.70	1.42	2.06	1.72	1.43	2.08
Previously assessed	0.56	0.49	0.65	0.57	0.50	0.65
Insurance	0.54	0.41	0.71	0.53	0.39	0.68
Referred	2.04	1.81	2.29	2.03	1.81	2.28
Diagnosis of heart disease	8.00	6.90	9.29	8.10	6.98	9.40
Reason for visit	5.60	4.90	6.40	5.61	4.91	6.42
Age	1	1	1.01			
Sex	0.92	0.84	1.02			
Race						
White	0.89	0.72	1.13			
Black	1.06	0.82	1.38			
Tobacco use	0.91	0.79	1.06			
Diabetes	0.97	0.84	1.11			
Obesity	1.16	0.99	1.34			
COPD	1.18	0.98	1.42			

OR, odds ratio; CI, confidence interval; CEBVD, cerebrovascular disease/history of stroke or transient ischemic attack; CHF, congestive heart failure; CAD, coronary artery disease/ischemic heart disease/history of myocardial infarction; COPD, chronic obstructive pulmonary disease; MSA, metropolitan statistical area.

### 3.3. Predictive accuracy and net benefit of the nomogram

**Figure 4:**
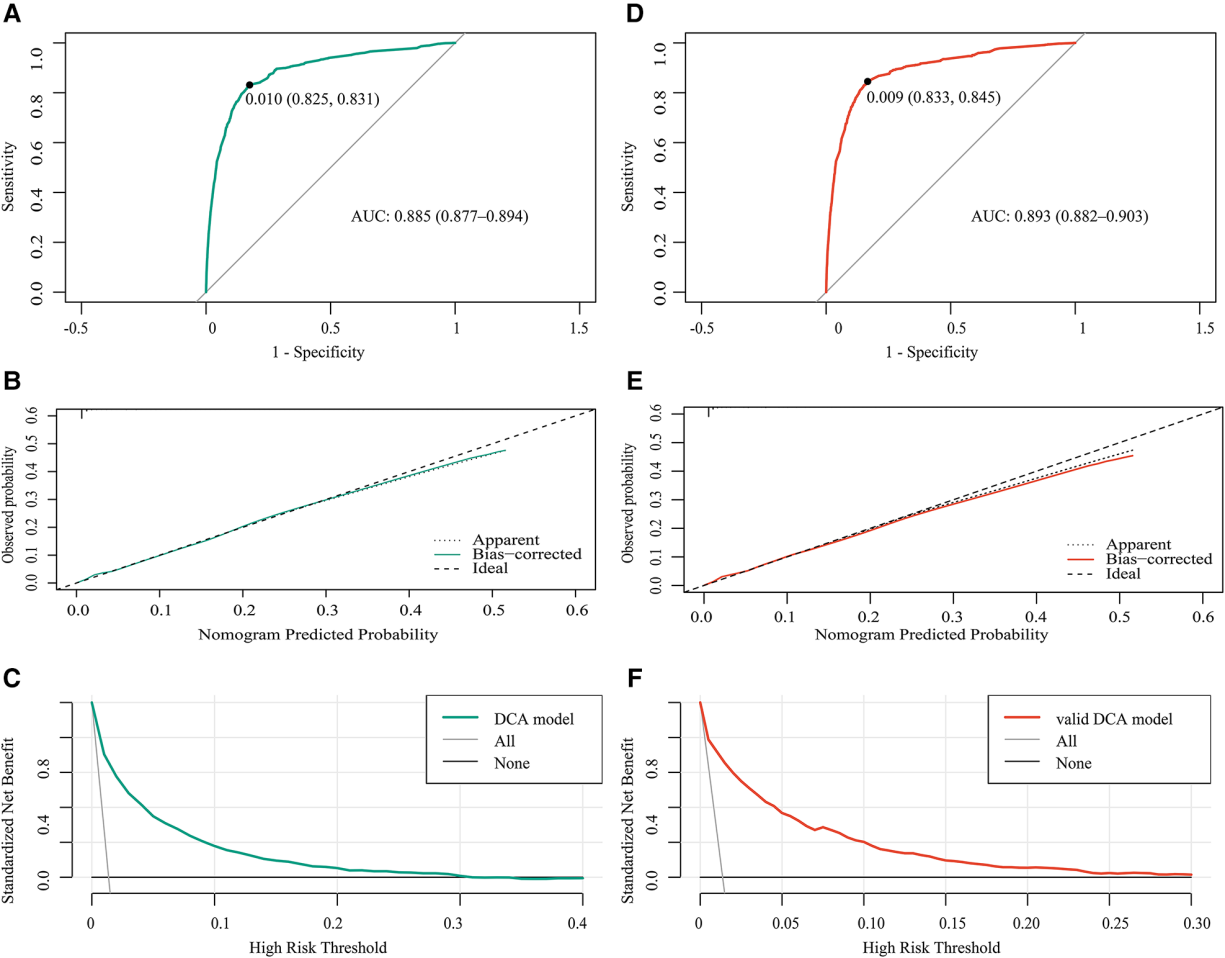
Receiver operating characteristic (ROC) curves for (**A**) the training cohort and (**D**) the validation cohort. (**B**,**E**) Calibration curve for the predictive ability of the training cohort (**B**) and the validation cohort (**E**) (**C**,**F**) Decision curve analysis of the net benefit of the prediction model in the training cohort (**C**) and the validation cohort (**F**) AUC, area under the curve; DCA, decision curve analysis.

In the training cohort, the area under the curve (AUC) was 0.885 (Figure 4A), and the calibration curve was close to the ideal diagonal line (Figure 4B). Meanwhile, the DCA showed that the prediction model had a good net benefit (Figure 4C).

A total of 86,871 patients were used for the internal validation of the nomogram. The AUC was 0.893 (Figure 4D), indicating that the nomogram had good accuracy. The model also had good consistency and the calibration curve was close to the ideal diagonal line (Figure 4E). The DCA showed that the prediction model also had a good net benefit in the validation cohort (Figure 4F).

### 3.4. The effect of echocardiography on the prescription of new cardiovascular agents

The conditions of the two groups of patients (echocardiogram/no-echocardiogram) were significantly different (Table 1). After PSM, the baseline characteristics were well-balanced. There was a significant difference in new cardiovascular agents prescribed between the two groups (*P* < 0.01) (Table 3). The abovementioned data demonstrated that the nomogram has good predictive potential for echocardiogram prescription and further contributes to clinical decision-making.

**Table 3:** Differences in new cardiovascular agents prescribed between the two groups after propensity score matching.

Variable	Total (*n *= 10676)	No echocardiogram (*n *= 8007)	Echocardiogram (*n *= 2669)	*P*-value
Age mean ± SD	63.25 ± 16.40	63.32 ± 16.45	63.07 ± 16.25	0.51
Sex *n* (%)				0.99
Male	5702 (53.41)	4276 (53.40)	1426 (53.43%)	
Female	4974 (46.59)	3731 (46.60)	1243 (46.57%)	
Race *n* (%)				0.93
White	8941 (83.75)	6712 (83.83)	2229 (83.51)	
Black	1220 (11.43)	911 (11.38)	309 (11.58)	
Other	515 (4.82)	384 (4.80)	131 (4.91)	
Tobacco use *n* (%)				0.56
No	9242 (86.57)	6941 (86.69)	2301 (86.21)	
Yes	1434 (13.43)	1066 (13.31)	368 (13.79)	
Insurance *n* (%)				0.29
No	318 (2.98)	230 (2.87)	88 (3.30)	
Yes	10358 (97.02)	7777 (97.13)	2581 (96.70)	
Reason for visit *n* (%)				0.12
Other	2740 (25.67)	2024 (25.28)	716 (26.83)	
Heart disease-related	7936 (74.33)	5983 (74.72)	1953 (73.17)	
Referred *n* (%)				0.17
No	6807 (63.76)	5135 (64.13)	1672 (62.65)	
Yes	3869 (36.24)	2872 (35.87)	997 (37.35)	
Previously assessed *n* (%)				0.40
No	2379 (22.28)	1768 (22.08)	611 (22.89)	
Yes	8297 (77.72)	6239 (77.92)	2058 (77.11)	
Major reason for visit *n* (%)				0.73
Other	7530 (70.53)	5655 (70.63)	1875 (70.25)	
New problem	3146 (29.47)	2352 (29.37)	794 (29.75)	
Diagnosis of heart disease *n* (%)				0.97
No	3013 (28.22)	2261 (28.24)	752 (28.18)	
Yes	7663 (71.78)	5746 (71.76)	1917 (71.82)	
CEBVD *n* (%)				0.16
no	10030 (93.95)	7538 (94.14)	2492 (93.37)	
yes	646 (6.05)	469 (5.86)	177 (6.63)	
CHF *n* (%)				0.11
No	9805 (91.84)	7374 (92.09)	2431 (91.08)	
Yes	871 (8.16)	633 (7.91)	238 (8.92)	
CAD *n* (%)				0.48
No	7905 (74.04)	5943 (74.22)	1962 (73.51)	
Yes	2771 (25.96)	2064 (25.78)	707 (26.49)	
Diabetes *n* (%)				0.60
No	8480 (79.43)	6350 (79.31)	2130 (79.81)	
Yes	2196 (20.57)	1657 (20.69)	539 (20.19)	
Hyperlipidemia *n* (%)				0.58
No	6201 (58.08)	4638 (57.92)	1563 (58.56)	
Yes	4475 (41.92)	3369 (42.08)	1106 (41.44)	
Hypertension *n* (%)				0.08
No	3980 (37.28)	2947 (36.81)	1033 (38.70)	
Yes	6696 (62.72)	5060 (63.19)	1636 (61.30)	
Obesity *n* (%)				0.60
No	9206 (86.23)	6896 (86.12)	2310 (86.55)	
Yes	1470 (13.77)	1111 (13.88)	359 (13.45)	
COPD				0.92
No	9867 (92.42)	7402 (92.44)	2465 (92.36)	
Yes	809 (7.58)	605 (7.56)	204 (7.64)	
MSA *n* (%)				0.55
No	794 (7.44)	588 (7.34)	206 (7.72)	
Yes	9882 (92.56)	7419 (92.66)	2463 (92.28)	
NCAP *n* (%)				<0.01
No	9459 (88.60)	7179 (89.66)	2280 (85.43)	
Yes	1217 (11.40)	828 (10.34)	389 (14.57)	

CEBVD, cerebrovascular disease/history of stroke or transient ischemic attack; CHF, congestive heart failure; CAD, coronary artery disease/ischemic heart disease/history of myocardial infarction; MSA, metropolitan statistical area; NCAP, new cardiovascular agents prescribed; COPD, chronic obstructive pulmonary disease.

## 4. Discussion

Our study is the first to develop a model for predicting echocardiography prescriptions based on outpatient variables. Hypertension, CHF, CEBVD, hyperlipidemia, CAD, major reason for visit, MSA, previously assessed, insurance, referred, diagnosis of cardiac disease, and reason for the visit were found to be predictors for the prescription of echocardiography. Echocardiography is an important test to assess heart structure and function. Based on our findings, echocardiography prescription was associated with the use of cardiovascular agents. Echocardiography imaging of cardiac structure and function can help guide patient management.

To reduce healthcare costs for outpatients with hypertension, especially the newly diagnosed, key aspects of the visit include determining the status of blood pressure control, medication use, and screening for complications when needed (12). To our surprise, based on our findings, hypertension was not a predictive factor in the prescription of an echocardiogram. In addition, according to the Appropriate Use Criteria for echocardiography guidelines (13), ordering an echocardiogram for the initial evaluation of suspected hypertensive heart disease is generally acceptable and reasonable. Prescription of echocardiography for routine evaluation of patients with systemic hypertension without suspected hypertensive heart disease or re-evaluation of a patient with known hypertensive heart disease without a change in clinical status is generally not acceptable or a reasonable approach. This could also explain why the existence of HBP in this database adds no value to the nomogram. Maybe these patients recently had an echocardiogram in the emergency room and did not require another one in the outpatient department (14). Among the general population, but particularly among patients with cardiovascular heart disease, hyperlipidemia is consistently associated with worse outcomes, including cardiovascular events, heart failure, and even death (15–17). Elevated residual cholesterol levels significantly improve myocardial infarction and ischemic heart disease risk prediction and are associated with an increased risk of cardiac death in patients with acute coronary syndromes (18, 19). Echocardiography is an excellent tool for monitoring therapy and has been proven to be useful in risk stratification upon presentation to the emergency ward, prior hospital discharge, and among outpatients with stable coronary heart disease, even in the absence of self-reported angina (7, 20). CAD, hyperlipidemia, and hypertension were all found to be independent predictors of echocardiography prescription in our study. This finding could be related to the necessity for echocardiography to evaluate the conditions of patients with CAD, and exclude cardiovascular disease in patients with hyperlipidemia.

Early referral among outpatients, especially when the cause for the visit is a new concern, is associated with better outcomes, fewer patient disputes, and reduced medical costs (21–25). It is also more conducive to further follow-up, and specialists can provide more appropriate treatment to previously assessed patients, minimizing the need for unnecessary additional examinations. In our study, both a new concern for the visit and a referred visit added points to the nomogram, indicating a need for an echocardiography.

We discovered that geographic and economic disparities exist in the use of echocardiography. Different living spaces correspond to distinct lifestyles and medical behaviors. In our study, patients from MSAs, with adding points in the nomogram model, were more likely to be prescribed an echocardiography. Other studies have shown geographic disparities in medication use or quality of care among patients (26, 27). Numerous studies have reported that, for both inpatients and outpatients, economic status, access to medical insurance, and the type and coverage of the insurance are all closely related to the treatment plan and adherence (28–30). Our analysis revealed an unexpected predictor. With the addition of a point, patients with no insurance were more likely to be prescribed an echocardiogram. The reasons for this are unclear. Nearly one in four Medicare beneficiaries received an echocardiogram within a given year (31). On average, the median rate of echocardiography (after the index study) was 0.72 studies per person per year, which were 100% Medicare fee-for-service claims (32). Maybe these patients recently had an echocardiogram in the emergency room and did not require another one in the outpatient department.

When integrated with clinical and ECG findings in an outpatient setting, echocardiography could be useful for the identification of ischemic cardiomyopathy when the reason for the visit was related to heart disease, or when cardiac disease was considered the initial diagnosis. Echocardiography, in conjunction with coronary angiography or computed tomographic coronary angiography, aids in the accurate detection of coronary heart disease. In the Appropriate Use Criteria for echocardiography guidelines, echocardiography is appropriate for the initial evaluation of patients with myocardial ischemia/infarction, valve disease, heart failure, cardiomyopathy, structure, and function, or for re-evaluation of these patients with a change in clinical status. The assessment of inferior vena cava size and collapsibility using echocardiography should allow for the prediction of readmission risk in patients with heart failure, as inadequate assessment of volume status leading to persistent congestion is an important contributing factor to rehospitalization (7–9, 33). Hyperlipidemia is strongly associated with cerebrovascular disease and coronary artery disease (34–36). The severity of carotid artery stenosis is significantly correlated with the extent of coronary artery disease, while the burden of carotid plaque is a strong predictor of cardiac death and MACE in patients with coronary artery disease (37–39). Therefore, the diagnosis of heart disease and the reason for visits related to heart disease are major predictors for the prescription of echocardiography.

The benefit of echocardiography over other imaging modalities is that it is widely available, inexpensive, and a bedside procedure. Echocardiography is usually the preferred method for the assessment of the size, segmental wall motion, and function of the left ventricle, the hemodynamics of the valve, and pulmonary pressure. Besides, it is also a useful tool in the diagnosis of myocardial infarction, heart failure, and congenital heart disease (7–9, 40). Advanced echocardiography (speckle tracking echocardiography and 3D echocardiography vs. 2D conventional echocardiography) is becoming more widely available and it may influence therapeutic decisions even more than conventional approaches (41, 42). Based on our findings, echocardiography prescription was related to the use of cardiovascular agents. Physicians may reevaluate the health state of the patients and devise a new medical plan due to the new echocardiography results.

Our study aims at providing clinical practice data on echocardiography in a real-world setting. We could provide information about the disparity between actual utilizations and guidelines and actual outpatient practice. Currently, echocardiogram prescription, according to the guidelines (43–46), should be clinically driven, until additional data about the use of this technology becomes available.

Our study had several limitations. First, a degree of internal bias was inevitable given the retrospective nature of the study. Due to the lack of follow-up data, we did not know whether the therapeutic changes following echocardiography had an impact on prognosis. Second, some potentially meaningful predictors, such as body mass index, were not assessed due to a lack of data (>30% missing). Third, we employed internal validation rather than external correction, thus the samples in this database can only be deemed representative of the population. Accordingly, we endeavor to undertake an external validation assessment using a different database in the future.

In conclusion, in this study, we demonstrated that hypertension, CHF, referred, CEBVD, hyperlipidemia, CAD, major reason for visit, previously assessed, MSA, insurance, diagnosis, and reason for the visit were predictors of prescribing echocardiography in outpatients, and built a characteristic nomogram to provide physicians with an intuitive tool for practical prediction. Our internal validation confirmed the good accuracy and conformity of the model, along with its net benefit. For each outpatient, a higher total score reflected a greater possibility of being prescribed an echocardiogram. The echocardiogram group was more likely to be prescribed new cardiovascular agents. These findings may contribute to providing information about the gap between actual utilizations and guidelines and the actual outpatient practice, as well as meeting the needs of outpatients.

## Data Availability

The datasets presented in this study can be found in online repositories. The names of the repository/repositories and accession number(s) can be found in the article/**Supplementary Material**.
