# Supplementary material for: A nomogram for predicting echocardiogram prescription in outpatients: an analysis of the NAMCS database
Source: Front Cardiovasc Med. 2023 Oct 16;10:1183504. doi: 10.3389/fcvm.2023.1183504 (PMC10613676; doi:10.3389/fcvm.2023.1183504)
Supplement: Supplementary file 1 [file Table1.docx]

Supplement Table S1. Characteristics at baseline between the echocardiogram and the no echocardiogram groups in pregnancy patients.

|  | Total  n=10271 | No echocardiogram n=10225 | Echocardiogram n=46 | *P*-value |
| --- | --- | --- | --- | --- |
| Age mean ± SD | 29.08 (6.64) | 29.07 (6.64) | 30.07 (6.62) | 0.31 |
| Race *n* (%) |  |  |  | 0.01 |
| white | 8055 (78.42) | 8011 (78.35) | 44 (95.65) |  |
| black | 1427 (13.89) | 1426 (13.95) | 1 (2.17) |  |
| other | 789 (7.68) | 788 (7.71) | 1 (2.17) |  |
| Tobacco use *n* (%) |  |  |  | 0.78 |
| no | 9135 (88.94) | 9093 (88.93) | 42 (91.30) |  |
| yes | 1136 (11.06) | 1132 (11.07) | 4 (8.70) |  |
| Insurance *n* (%) |  |  |  | 0.23 |
| no | 9762 (95.04) | 9720 (95.06) | 42 (91.30) |  |
| yes | 509 (4.96) | 505 (4.94) | 4 (8.70) |  |
| Reason for visit *n* (%) |  |  |  | <0.01 |
| other | 9623 (93.69) | 9597 (93.86) | 26 (56.52) |  |
| heart disease-related | 648 (6.31) | 628 (6.14) | 20 (43.48) |  |
| Referred *n* (%) |  |  |  | <0.01 |
| no | 8590 (83.63) | 8580 (83.91) | 10 (21.74) |  |
| yes | 1681 (16.37) | 1645 (16.09) | 36 (78.26) |  |
| Previously assessed *n* (%) |  |  |  | <0.01 |
| no | 973 (9.47) | 954 (9.33) | 19 (41.30) |  |
| yes | 9298 (90.53) | 9271 (90.67) | 27 (58.70) |  |
| Major reason for visit *n* (%) |  |  |  | <0.01 |
| other | 9324 (90.78) | 9292 (90.88) | 32 (69.57) |  |
| new problem | 947 (9.22) | 933 (9.12) | 14 (30.43) |  |
| Diagnosis of heart disease *n* (%) |  |  |  | <0.01 |
| no | 10161 (98.93) | 10125 (99.02) | 36 (78.26) |  |
| yes | 110 (1.07) | 100 (0.98) | 10 (21.74) |  |
| CEBVD *n* (%) |  |  |  | 0.99 |
| no | 10263 (99.92) | 10217 (99.92%) | 46 (100.00) |  |
| yes | 8 (0.08) | 8 (0.08) | 0 (0.00) |  |
| COPD n (%) |  |  |  | 0.09 |
| no | 10249 (99.79) | 10204 (99.79) | 45 (97.83) |  |
| yes | 22 (0.21) | 21 (0.21) | 1 (2.17) |  |
| CHF *n* (%) |  |  |  | 0.04 |
| no | 10262 (99.91) | 10217 (99.92) | 45 (97.83) |  |
| yes | 9 (0.09) | 8 (0.08) | 1 (2.17) |  |
| CAD *n* (%) |  |  |  | 0.99 |
| no | 10253 (99.82) | 10207 (99.82) | 46 (100.00) |  |
| yes | 18 (0.18) | 18 (0.18) | 0 (0.00) |  |
| Diabetes *n* (%) |  |  |  | 0.44 |
| no | 9853 (95.93) | 9810 (95.94) | 43 (93.48) |  |
| yes | 418 (4.07) | 415 (4.06) | 3 (6.52) |  |
| Hyperlipidemia *n* (%) |  |  |  | 0.29 |
| no | 10194 (99.25) | 10149 (99.26) | 45 (97.83) |  |
| yes | 77 (0.75) | 76 (0.74) | 1 (2.17) |  |
| Hypertension *n* (%) |  |  |  | 0.05 |
| no | 9829 (95.70) | 9788 (95.73) | 41 (89.13) |  |
| yes | 442 (4.30) | 437 (4.27) | 5 (10.87) |  |
| Obesity *n* (%) |  |  |  | 0.07 |
| no | 9778 (95.20) | 9737 (95.23) | 41 (89.13) |  |
| yes | 493 (4.80) | 488 (4.77) | 5 (10.87) |  |
| MSA *n* (%) |  |  |  | 0.23 |
| no | 1104 (10.75) | 1102 (10.78) | 2 (4.35) |  |
| yes | 9167 (89.25) | 9123 (89.22) | 44 (95.65) |  |

CEBVD: cerebrovascular disease/history of stroke or transient ischemic attack; CHF: congestive heart failure; CAD: coronary artery disease/ischemic heart disease/history of myocardial infarction; MSA: metropolitan statistical area; COPD: chronic obstructive pulmonary disease.

Table S2. The classification and code of cardiovascular medication in the NAMCS database.

| Level 1 | | Level 2 | | Level 3 | |
| --- | --- | --- | --- | --- | --- |
| Code ID | Category name | Code ID | Category name | Code ID | Category name |
| 040 | cardiovascular agents | 041 | agents for hypertensive emergencies |  |  |
| 040 | cardiovascular agents | 042 | angiotensin converting enzyme inhibitors |  |  |
| 040 | cardiovascular agents | 043 | antiadrenergic agents, peripherally acting |  |  |
| 040 | cardiovascular agents | 044 | antiadrenergic agents, centrally acting |  |  |
| 040 | cardiovascular agents | 045 | antianginal agents |  |  |
| 040 | cardiovascular agents | 046 | antiarrhythmic agents | 385 | group I antiarrhythmics |
| 040 | cardiovascular agents | 046 | antiarrhythmic agents | 386 | group II antiarrhythmics |
| 040 | cardiovascular agents | 046 | antiarrhythmic agents | 387 | group III antiarrhythmics |
| 040 | cardiovascular agents | 046 | antiarrhythmic agents | 388 | group IV antiarrhythmics |
| 040 | cardiovascular agents | 046 | antiarrhythmic agents | 389 | group V antiarrhythmics |
| 040 | cardiovascular agents | 047 | beta-adrenergic blocking agents | 274 | cardioselective beta blockers |
| 040 | cardiovascular agents | 047 | beta-adrenergic blocking agents | 275 | non-cardioselective beta blockers |
| 040 | cardiovascular agents | 048 | calcium channel blocking agents |  |  |
| 040 | cardiovascular agents | 049 | diuretics | 154 | loop diuretics |
| 040 | cardiovascular agents | 049 | diuretics | 155 | potassium-sparing diuretics |
| 040 | cardiovascular agents | 049 | diuretics | 156 | thiazide and thiazide-like diuretics |
| 040 | cardiovascular agents | 049 | diuretics | 157 | carbonic anhydrase inhibitors |
| 040 | cardiovascular agents | 049 | diuretics | 158 | miscellaneous diuretics |
| 040 | cardiovascular agents | 050 | inotropic agents |  |  |
| 040 | cardiovascular agents | 051 | miscellaneous cardiovascular agents |  |  |
| 040 | cardiovascular agents | 052 | peripheral vasodilators |  |  |
| 040 | cardiovascular agents | 053 | vasodilators |  |  |
| 040 | cardiovascular agents | 054 | vasopressors |  |  |
| 040 | cardiovascular agents | 055 | antihypertensive combinations |  |  |
| 040 | cardiovascular agents | 056 | angiotensin II inhibitors |  |  |
| 040 | cardiovascular agents | 303 | agents for pulmonary hypertension |  |  |
| 040 | cardiovascular agents | 319 | vasopressin antagonists |  |  |
| 040 | cardiovascular agents | 325 | sclerosing agents |  |  |
| 040 | cardiovascular agents | 340 | aldosterone receptor agonists |  |  |
| 040 | cardiovascular agents | 342 | renin inhibitors |  |  |
| 040 | cardiovascular agents | 396 | prostaglandin D2 antagonists |  |  |
| 040 | cardiovascular agents | 430 | anticholinergic chronotropic agents |  |  |
| 040 | cardiovascular agents | 433 | catecholamines |  |  |
| 081 | coagulation modifiers | 082 | anticoagulants | 261 | heparins |
| 081 | coagulation modifiers | 082 | anticoagulants | 262 | coumarins and indandiones |
| 081 | coagulation modifiers | 082 | anticoagulants | 283 | thrombin inhibitors |
| 081 | coagulation modifiers | 082 | anticoagulants | 285 | factor Xa inhibitors |
| 081 | coagulation modifiers | 083 | antiplatelet agents | 211 | platelet aggregation inhibitors |
| 081 | coagulation modifiers | 083 | antiplatelet agents | 212 | glycoprotein platelet inhibitors |
| 081 | coagulation modifiers | 083 | antiplatelet agents | 463 | protease-activated receptor-1 antagonists |
| 358 | metabolic agents | 019 | antihyperlipidemic agents | 173 | HMG-CoA reductase inhibitors |
| 358 | metabolic agents | 019 | antihyperlipidemic agents | 174 | miscellaneous antihyperlipidemic agents |
| 358 | metabolic agents | 019 | antihyperlipidemic agents | 241 | fibric acid derivatives |
| 358 | metabolic agents | 019 | antihyperlipidemic agents | 252 | bile acid sequestrants |
| 358 | metabolic agents | 019 | antihyperlipidemic agents | 316 | cholesterol absorption inhibitors |
| 358 | metabolic agents | 019 | antihyperlipidemic agents | 317 | antihyperlipidemic combinations |

Table S3 Characteristics at baseline between the echocardiogram and the no echocardiogram groups in year 2014–2019.

| Variable | Total^*^ (n=54052) | No echocardiogram (n=53475) | Echocardiogram (n=577) | *P*-value |
| --- | --- | --- | --- | --- |
| Age mean ± SD | 57.20 (17.97) | 57.14 (17.98) | 62.69 (16.25) | <0.01 |
| Sex *n* (%) |  |  |  | 0.60 |
| male | 31074 (57.49%) | 30749 (57.50%) | 325 (56.33%) |  |
| female | 22978 (42.51%) | 22726 (42.50%) | 252 (43.67%) |  |
| Race *n* (%) |  |  |  | <0.01 |
| white | 46905 (86.78%) | 46435 (86.83%) | 470 (81.46%) |  |
| black | 4693 (8.68%) | 4605 (8.61%) | 88 (15.25%) |  |
| other | 2454 (4.54%) | 2435 (4.55%) | 19 (3.29%) |  |
| Tobacco use *n* (%) |  |  |  | 0.11 |
| no | 46891 (86.75%) | 46377 (86.73%) | 514 (89.08%) |  |
| yes | 7161 (13.25%) | 7098 (13.27%) | 63 (10.92%) |  |
| Insurance *n* (%) |  |  |  | <0.01 |
| no | 50856 (94.09%) | 50293 (94.05%) | 563 (97.57%) |  |
| yes | 3196 (5.91%) | 3182 (5.95%) | 14 (2.43%) |  |
| Reason for visit *n* (%) |  |  |  | <0.01 |
| other | 46667 (86.34%) | 46491 (86.94%) | 176 (30.50%) |  |
| heart disease-related | 7385 (13.66%) | 6984 (13.06%) | 401 (69.50%) |  |
| Referred *n* (%) |  |  |  | <0.01 |
| no | 37358 (69.11%) | 37029 (69.25%) | 329 (57.02%) |  |
| yes | 16694 (30.89%) | 16446 (30.75%) | 248 (42.98%) |  |
| Previously assessed |  |  |  | <0.01 |
| no | 12120 (22.42%) | 11934 (22.32%) | 186 (32.24%) |  |
| yes | 41932 (77.58%) | 41541 (77.68%) | 391 (67.76%) |  |
| Major reason for visit *n* (%) |  |  |  | 0.35 |
| other | 36164 (66.91%) | 35767 (66.89%) | 397 (68.80%) |  |
| new problem | 17888 (33.09%) | 17708 (33.11%) | 180 (31.20%) |  |
| Diagnosis of heart disease *n* (%) |  |  |  | <0.01 |
| no | 50264 (92.99%) | 50025 (93.55%) | 239 (41.42%) |  |
| yes | 3788 (7.01%) | 3450 (6.45%) | 338 (58.58%) |  |
| CEBVD *n* (%) |  |  |  | <0.01 |
| no | 52814 (97.71%) | 52278 (97.76%) | 536 (92.89%) |  |
| yes | 1238 (2.29%) | 1197 (2.24%) | 41 (7.11%) |  |
| CHF *n* (%) |  |  |  | <0.01 |
| no | 53122 (98.28%) | 52598 (98.36%) | 524 (90.81%) |  |
| yes | 930 (1.72%) | 877 (1.64%) | 53 (9.19%) |  |
| CAD *n* (%) |  |  |  | <0.01 |
| no | 50316 (93.09%) | 49924 (93.36%) | 392 (67.94%) |  |
| yes | 3736 (6.91%) | 3551 (6.64%) | 185 (32.06%) |  |
| Diabetes *n* (%) |  |  |  | <0.01 |
| no | 46486 (86.00%) | 46037 (86.09%) | 449 (77.82%) |  |
| yes | 7566 (14.00%) | 7438 (13.91%) | 128 (22.18%) |  |
| Hyperlipidemia *n* (%) |  |  |  | <0.01 |
| no | 43479 (80.44%) | 43175 (80.74%) | 304 (52.69%) |  |
| yes | 10573 (19.56%) | 10300 (19.26%) | 273 (47.31%) |  |
| Hypertension *n* (%) |  |  |  | <0.01 |
| no | 36304 (67.16%) | 36092 (67.49%) | 212 (36.74%) |  |
| yes | 17748 (32.84%) | 17383 (32.51%) | 365 (63.26%) |  |
| Obesity *n* (%) |  |  |  | <0.01 |
| no | 49990 (92.49%) | 49500 (92.57%) | 490 (84.92%) |  |
| yes | 4062 (7.51%) | 3975 (7.43%) | 87 (15.08%) |  |
| COPD *n* (%) |  |  |  | <0.01 |
| no | 52022 (96.24%) | 51489 (96.29%) | 533 (92.37%) |  |
| yes | 2030 (3.76%) | 1986 (3.71%) | 44 (7.63%) |  |
| CKD *n* (%) |  |  |  | <0.01 |
| no | 52583 (97.28%) | 52038 (97.31%) | 545 (94.45%) |  |
| yes | 1469 (2.72%) | 1437 (2.69%) | 32 (5.55%) |  |
| ESRD *n* (%) |  |  |  | 0.99 |
| no | 53922 (99.76%) | 53346 (99.76%) | 576 (99.83%) |  |
| yes | 130 (0.24%) | 129 (0.24%) | 1 (0.17%) |  |
| MSA *n* (%) |  |  |  | <0.01 |
| no | 4930 (9.12%) | 4902 (9.17%) | 28 (4.85%) |  |
| yes | 49122 (90.88%) | 48573 (90.83%) | 549 (95.15%) |  |

CEBVD: cerebrovascular disease/history of stroke or transient ischemic attack; CHF: congestive heart failure; CAD: coronary artery disease/ischemic heart disease/history of myocardial infarction; MSA: metropolitan statistical area.; COPD: chronic obstructive pulmonary disease; CKD: chronic kidney disease; ESRD: end-stage renal disease.

*: Only 2014–2019 data were analyzed.

Table S4. Echocardiogram multivariable logistic regression model in the population 2014-2019.

| Variable | OR^*^[95%CI] | P value |
| --- | --- | --- |
| Age | 0.997(0.991~1.003) | 0.333 |
| Race |  |  |
| black | 1.476(1.146~1.88) | <0.05 |
| other | 0.704(0.422~1.105) | 0.15 |
| Insurance | 0.737(0.405~1.231) | 0.277 |
| Reason for visit | 6.175(4.816~7.966) | <0.05 |
| Referred | 1.727(1.406~2.116) | <0.05 |
| Previously assessed | 0.449(0.359~0.561) | <0.05 |
| Diagnosis of heart disease | 9.798(7.469~12.929) | <0.05 |
| CEBVD | 1.658(1.153~2.328) | <0.05 |
| CHF | 1.473(1.058~2.016) | <0.05 |
| CAD | 1.617(1.309~1.992) | <0.05 |
| Diabetes | 1.086(0.871~1.346) | 0.459 |
| Hyperlipidemia | 1.353(1.112~1.646) | <0.05 |
| Hypertension | 0.636(0.511~0.794) | <0.05 |
| Obesity | 1.133(0.878~1.446) | 0.327 |
| MSA | 1.759(1.212~2.659) | <0.05 |
| COPD | 1.124(0.794~1.553) | 0.495 |
| CKD | 0.713(0.475~1.036) | 0.088 |

CEBVD: cerebrovascular disease/history of stroke or transient ischemic attack; CHF: congestive heart failure; CAD: coronary artery disease/ischemic heart disease/history of myocardial infarction; MSA: metropolitan statistical area.; COPD: chronic obstructive pulmonary disease; CKD: chronic kidney disease.

*: Only 2014–2019 data were analyzed.

Table S5. Echocardiogram multivariable logistic regression model based on the results of the LASSO regression analysis in the population 2014-2019

| Variable | OR^*^[95%CI] | P value |
| --- | --- | --- |
| Age | 0.996(0.99~1.002) | 0.219 |
| female | 0.766(0.642~0.914) | <0.05 |
| Race |  |  |
| black | 1.454(1.128~1.856) | <0.05 |
| other | 0.703(0.421~1.104) | 0.149 |
| Tobacco use | 0.867(0.65~1.139) | 0.319 |
| Insurance | 0.763(0.419~1.278) | 0.339 |
| Reason for visit | 6.671(5.186~8.633) | <0.05 |
| Referred | 1.719(1.398~2.11) | <0.05 |
| Previously assessed | 0.497(0.394~0.627) | <0.05 |
| Major reason for visit | 1.492(1.216~1.825) | <0.05 |
| Diagnosis of heart disease | 9.669(7.369~12.764) | <0.05 |
| CEBVD | 1.69(1.174~2.375) | <0.05 |
| CHF | 1.505(1.079~2.063) | <0.05 |
| CAD | 1.709(1.381~2.111) | <0.05 |
| Diabetes | 1.101(0.882~1.366) | 0.387 |
| Hyperlipidemia | 1.387(1.14~1.69) | <0.05 |
| Hypertension | 0.646(0.518~0.806) | <0.05 |
| Obesity | 1.118(0.866~1.428) | 0.383 |
| MSA | 1.748(1.204~2.645) | <0.05 |
| COPD | 1.141(0.803~1.584) | 0.447 |
| CKD | 0.734(0.488~1.068) | 0.12 |

CEBVD: cerebrovascular disease/history of stroke or transient ischemic attack; CHF: congestive heart failure; CAD: coronary artery disease/ischemic heart disease/history of myocardial infarction; MSA: metropolitan statistical area.; COPD: chronic obstructive pulmonary disease; CKD: chronic kidney disease.

*: Only 2014–2019 data were analyzed.

Table S6. Characteristic at baseline between training cohort and validation cohort

| Variable | Total ( n=217178 ) | validation cohort ( n=86871 ) | training cohort ( n=130307 ） | P value |
| --- | --- | --- | --- | --- |
| Age n (%) | 55.86 (18.09) | 55.88 (18.08) | 55.86 (18.09) | 0.78 |
| Sex n (%) |  |  |  | 0.78 |
| male | 127168 (58.55) | 50899 (58.59) | 76269 (58.53) |  |
| female | 90010 (41.45) | 35972 (41.41) | 54038 (41.47) |  |
| Race n (%) |  |  |  | 0.86 |
| white | 186282 (85.77) | 74516 (85.78) | 111766 (85.77) |  |
| black | 20526 (9.45) | 8186 (9.42) | 12340 (9.47) |  |
| other | 10370 (4.77) | 4169 (4.80) | 6201 (4.76) |  |
| Tobacco Use n(%) |  |  |  | 0.04 |
| no | 182556 (84.06) | 72854 (83.86) | 109702 (84.19) |  |
| yes | 34622 (15.94) | 14017 (16.14) | 20605 (15.81) |  |
| Insurance n (%) |  |  |  | 0.69 |
| no | 18305 (8.43) | 7348 (8.46) | 10957 (8.41) |  |
| yes | 198873 (91.57) | 79523 (91.54) | 119350 (91.59) |  |
| Reason for visit n (%) |  |  |  | 0.73 |
| other | 178136 (82.02) | 71285 (82.06) | 106851 (82.00) |  |
| heart disease related | 39042 (17.98) | 15586 (17.94) | 23456 (18.00) |  |
| Referred n(%) |  |  |  | 0.55 |
| no | 157425 (72.49) | 62908 (72.42) | 94517 (72.53) |  |
| yes | 59753 (27.51) | 23963 (27.58) | 35790 (27.47) |  |
| Seen before n(%) |  |  |  | 0.56 |
| no | 43759 (20.15) | 17558 (20.21) | 26201 (20.11) |  |
| yes | 173419 (79.85) | 69313 (79.79) | 104106 (79.89) |  |
| Major reason for visit n(%) |  |  |  | 0.48 |
| other | 142923 (65.81) | 57092 (65.72) | 85831 (65.87) |  |
| new problem | 74255 (34.19) | 29779 (34.28) | 44476 (34.13) |  |
| Diagnosis of heart disease n(%) |  |  |  | 0.11 |
| no | 185562 (85.44) | 74353 (85.59) | 111209 (85.34) |  |
| yes | 31616 (14.56) | 12518 (14.41) | 19098 (14.66) |  |
| CEBVD n(%) |  |  |  | 0.85 |
| no | 212131 (97.68) | 84859 (97.68) | 127272 (97.67) |  |
| yes | 5047 (2.32) | 2012 (2.32) | 3035 (2.33) |  |
| CHF n(%) |  |  |  | 0.43 |
| no | 212864 (98.01) | 85171 (98.04) | 127693 (97.99) |  |
| yes | 4314 (1.99) | 1700 (1.96) | 2614 (2.01) |  |
| CAD n(%) |  |  |  | 0.54 |
| no | 205643 (94.69) | 82289 (94.73) | 123354 (94.66) |  |
| yes | 11535 (5.31) | 4582 (5.27) | 6953 (5.34) |  |
| Diabetes n(%) |  |  |  | 0.64 |
| no | 187841 (86.49) | 75099 (86.45) | 112742 (86.52) |  |
| yes | 29337 (13.51) | 11772 (13.55) | 17565 (13.48) |  |
| Hyperlipidemia n (%) |  |  |  | 0.25 |
| no | 177720 (81.83) | 71189 (81.95) | 106531 (81.75) |  |
| yes | 39458 (18.17) | 15682 (18.05) | 23776 (18.25) |  |
| Hypertension n(%) |  |  |  | 0.18 |
| no | 148339 (68.30) | 59478 (68.47) | 88861 (68.19) |  |
| yes | 68839 (31.70) | 27393 (31.53) | 41446 (31.81) |  |
| Obesity n(%) |  |  |  | 0.53 |
| no | 200580 (92.36) | 80193 (92.31) | 120387 (92.39) |  |
| yes | 16598 (7.64) | 6678 (7.69) | 9920 (7.61) |  |
| COPD n(%) |  |  |  | 0.10 |
| no | 207919 (95.74) | 83243 (95.82) | 124676 (95.68) |  |
| yes | 9259 (4.26) | 3628 (4.18) | 5631 (4.32) |  |
| MSA n(%) |  |  |  | 0.45 |
| no | 24159 (11.12) | 9609 (11.06) | 14550 (11.17) |  |
| yes | 193019 (88.88) | 77262 (88.94) | 115757 (88.83) |  |

CEBVD: cerebrovascular disease/history of stroke or transient ischemic attack; CHF: congestive heart failure; CAD: coronary artery disease/ischemic heart disease/history of myocardial infarction; MSA: metropolitan statistical area; COPD: chronic obstructive pulmonary disease.

Table S7. The univariate logistic regression analysis of the characteristics in the training cohort

| Variable | Total ( n=130307 ) | no-echocardiogram （ n=128523） | echocardiogram （ n=1784 ） | OR[95%CI] | P value |
| --- | --- | --- | --- | --- | --- |
| Age n (%) | 55.86 (18.09) | 55.76 (18.09) | 62.74 (16.44) | 1.02 [1.02;1.03] | <0.01 |
| Sex n (%) |  |  |  |  | <0.01 |
| male | 76269 (58.53) | 75302 (58.59) | 967 (54.20) | Ref. |  |
| female | 54038 (41.47) | 53221 (41.41) | 817 (45.80) | 1.20 [1.09;1.31] |  |
| Race n (%) |  |  |  |  | <0.01 |
| white | 111766 (85.77) | 110294 (85.82) | 1472 (82.51) | Ref. |  |
| black | 12340 (9.47) | 12118 (9.43) | 222 (12.44) | 1.37 [1.19;1.58] |  |
| other | 6201 (4.76) | 6111 (4.75) | 90 (5.04) | 1.11 [0.89;1.36] |  |
| Tobacco use n(%) |  |  |  |  | 0.01 |
| no | 109702 (84.19) | 108158 (84.15) | 1544 (86.55) | Ref. |  |
| yes | 20605 (15.81) | 20365 (15.85) | 240 (13.45) | 0.83 [0.72;0.95] |  |
| Insurance n (%) |  |  |  |  | <0.01 |
| no | 10957 (8.41) | 10901 (8.48) | 56 (3.14) | 0.35 [0.27;0.45] |  |
| yes | 119350 (91.59) | 117622 (91.52) | 1728 (96.86) | Ref. |  |
| Reason for visit n (%) |  |  |  |  | <0.01 |
| other | 106851 (82.00) | 106411 (82.80) | 440 (24.66) | Ref. |  |
| heart disease related | 23456 (18.00) | 22112 (17.20) | 1344 (75.34) | 14.7 [13.2;16.4] |  |
| Referred n(%) |  |  |  |  | <0.01 |
| no | 94517 (72.53) | 93506 (72.75) | 1011 (56.67) | Ref. |  |
| yes | 35790 (27.47) | 35017 (27.25) | 773 (43.33) | 2.04 [1.86;2.24] |  |
| Seen before n(%) |  |  |  |  | <0.01 |
| no | 26201 (20.11) | 25664 (19.97) | 537 (30.10) | Ref. |  |
| yes | 104106 (79.89) | 102859 (80.03) | 1247 (69.90) | 0.58 [0.52;0.64] |  |
| Major reason for visit n(%) |  |  |  |  | 0.70 |
| other | 85831 (65.87) | 84664 (65.87) | 1167 (65.41) | Ref. |  |
| new problem | 44476 (34.13) | 43859 (34.13) | 617 (34.59) | 1.02 [0.92;1.13] |  |
| Diagnosis of heart disease n(%) |  |  |  |  | <0.01 |
| no | 111209 (85.34) | 110749 (86.17) | 460 (25.78) | Ref. |  |
| yes | 19098 (14.66) | 17774 (13.83) | 1324 (74.22) | 17.9 [16.1;20.0] |  |
| CEBVD n(%) |  |  |  |  | <0.01 |
| no | 127272 (97.67) | 125617 (97.74) | 1655 (92.77) | Ref. |  |
| yes | 3035 (2.33) | 2906 (2.26) | 129 (7.23) | 3.37 [2.80;4.03] |  |
| CHF n(%) |  |  |  |  | <0.01 |
| no | 127693 (97.99) | 126071 (98.09) | 1622 (90.92) | Ref. |  |
| yes | 2614 (2.01) | 2452 (1.91) | 162 (9.08) | 5.14 [4.34;6.05] |  |
| CAD n(%) |  |  |  |  | <0.01 |
| no | 123354 (94.66) | 122047 (94.96) | 1307 (73.26) | Ref. |  |
| yes | 6953 (5.34) | 6476 (5.04) | 477 (26.74) | 6.88 [6.17;7.66] |  |
| Diabetes n(%) |  |  |  |  | <0.01 |
| no | 112742 (86.52) | 111322 (86.62) | 1420 (79.60) | Ref. |  |
| yes | 17565 (13.48) | 17201 (13.38) | 364 (20.40) | 1.66 [1.48;1.86] |  |
| Hyperlipidemia n (%) |  |  |  |  | <0.01 |
| no | 106531 (81.75) | 105481 (82.07) | 1050 (58.86) | Ref. |  |
| yes | 23776 (18.25) | 23042 (17.93) | 734 (41.14) | 3.20 [2.91;3.52] |  |
| Hypertension n(%) |  |  |  |  | <0.01 |
| no | 88861 (68.19) | 88154 (68.59) | 707 (39.63) | Ref. |  |
| yes | 41446 (31.81) | 40369 (31.41) | 1077 (60.37) | 3.33 [3.02;3.66] |  |
| Obesity n(%) |  |  |  |  | <0.01 |
| no | 120387 (92.39) | 118841 (92.47) | 1546 (86.66) | Ref. |  |
| yes | 9920 (7.61) | 9682 (7.53) | 238 (13.34) | 1.89 [1.64;2.17] |  |
| COPD n(%) |  |  |  |  |  |
| no | 124676 (95.68) | 123041 (95.73) | 1635 (91.65) | Ref |  |
| yes | 5631 (4.32) | 5482 (4.27) | 149 (8.35) | 2.05 [1.72;2.42] |  |
| MSA: |  |  |  |  | <0.01 |
| no | 14550 (11.17) | 14422 (11.22) | 128 (7.17) | Ref. |  |
| yes | 115757 (88.83) | 114101 (88.78) | 1656 (92.83) | 1.63 [1.37;1.97] |  |

OR: odds ratio; CI: confidence interval; CEBVD: cerebrovascular disease/history of stroke or transient ischemic attack; CHF: congestive heart failure; CAD: coronary artery disease/ischemic heart disease/history of myocardial infarction; MSA: metropolitan statistical area; COPD: chronic obstructive pulmonary disease.
